# Annual acknowledgement of manuscript reviewers

**DOI:** 10.1186/1475-2840-13-18

**Published:** 2014-01-30

**Authors:** Alexander Tenenbaum, Enrique Fisman

**Affiliations:** 1 Cardiac Rehabilitation Institute, Sheba Medical Center, Tel-Hashomer 52621, Israel; 2 Sackler Faculty of Medicine, Tel-Aviv University, Tel-Aviv 69978, Israel

## Abstract

**Contributing reviewers:**

The Editors of Cardiovascular Diabetology wish to thank all reviewers, both Editorial Board Members and external referees, who contributed to the journal in 2013 (Volume 12), and whose enthusiastic support in providing the essential service of peer review continues to improve the scientific quality of the journal and enable its goals to be achieved.

## 

Ellen Aasum

Norway

Shefali Agrawal

India

Sofia Ahmed

Canada

Sayer Al-Azzam

Jordan

Markus Sallman Almen

Sweden

Ezequiel Álvarez

Spain

Rosa Amoroso

Italy

Ethan J. Anderson

USA

Francesco Andreozzi

Italy

Alessandro Antenore

Italy

Teresa Arora

UK

Kei Asayama

Japan

Ambika Ashraf

USA

Yoshimasa Aso

Japan

Fatmahan Atalar

UK

Angelo Avogaro

Italy

Julio E. Ayala

USA

Jan Azarov

Russia

Ok-Nam Bae

South Korea

Babu Balagopal

USA

Maria L. Balestrieri

Italy

Yasuko K. K. Bando

Japan

Weike Bao

USA

Marta Baranowska-Kuczko

Poland

Darius Batulevicius

Lithuania

Christophe Beauloye

Belgium

Vitantonio Di Bello

Italy

Partha Bhattacharjee

USA

Helene von Bibra

Germany

Benjamin T. Bikman

USA

Federico Biscetti

Italy

Dirk Blom

South Africa

Mona Boaz

Israel

Yuri Bobryshev

Australia

Mauro Boronat

Spain

Luisa-María Botella

Spain

Peter Bramlage

Germany

Patrice Brassard

Canada

Helmut Brath

Austria

Charissa van den Brom

Netherlands

Claudia Bruedigam

Australia

Antonio Brunetti

Italy

Roland Buettner

Germany

Monika Buraczynska

Poland

Paul Burchardt

Poland

Lu Cai

USA

Zheqing P. Cai

USA

Philip Calder

UK

Sebastián D. Calligaris

Chile

Amadou K. S. Camara

USA

Andrea Caporali

UK

Carmine Cardillo

Italy

Claudia R. Cardoso

Brazil

Eugenia Carvalho

Portugal

Chiara Caselli

Italy

Oscar Casis

Spain

Sule Cataltepe

USA

Jan Cederholm

Sweden

Antonio Ceriello

Spain

Daniela Cesselli

Italy

Matthew Cham

USA

Eric J. Belin de Chantemèle

USA

Nipon Chattipakorn

Thailand

Rima Chedid

Lebanon

Gopic Chellan

India

Li-ming Chen

China

Wenqiang Chen

China

Zheng Chen

USA

Juei-Tang Cheng

Taiwan

Min Cheng

China

Kazuo Chin

Japan

Sungsam Cho

Japan

Virasakdi Chongsuvivatwong

Thailand

Marco Ciccone

Italy

Figen A. Cicek

Turkey

Arrigo F. G. Cicero

Italy

Geraldine Clough

UK

Paolo Colonna

Italy

Daniel Conklin

USA

Alberto Corsini

Italy

Eliecer Coto

Spain

Amanda J. Cox

USA

Alberto Crottogini

Argentina

Tamas Csont

Hungary

Taixing Cui

USA

Jordi S. Dahl

Denmark

Hiroyuki Daida

Japan

Ravi Dasu

USA

Sean M. Davidson

UK

Sylvie Dejager

France

K. M. Demaris

USA

Gareth Denyer

Australia

Giuseppe Derosa

Italy

Neeraj N. Dhaun

UK

Bob Dobbins

USA

Lily Dong

USA

Luciano Drager

Brazil

Robin P. F. Dullaart

Netherlands

Magdalena Dvorakova

Czech Republic

Bernd Ebner

Germany

Igor Efimov

USA

Sonia Eiras

Spain

Carl Ekman

Sweden

Theodoros Eleftheriadis

Greece

Robert S. Elkeles

UK

Francisco J. Enguita

Portugal

Porat Erlich

USA

Joan C. Escola-Gil

Spain

Ricardo J. Esper

Argentina

Mahyar Etminan

Canada

Maximilian von Eynatten

Germany

Paola Failli

Italy

Vera Farah

Brazil

Massimo Federici

Italy

Giovanni Federico

Italy

Peter Ferdinandy

Hungary

Thomas Flagg

USA

Alessia Fornoni

USA

Nikolaos G. Frangogiannis

USA

Jeffrey Freeman

USA

Thomas Frese

Germany

Zhenhong Fu

China

Takaaki Fujii

Japan

Michiaki Fukui

Japan

Seiji Fukumoto

Japan

Frédéric Fumeron

France

Merrill Funk

USA

Baptist Gallwitz

Germany

Spiros D. D. Garbis

UK

Vishvas Garg

USA

Armen Y. Gasparyan

UK

Galia Gat-Yablonski

Israel

Asish Ghosh

USA

Richard Gilbert

Canada

Philippe Gillery

France

Carlo B. Giorda

Italy

Ilaria Giordani

Italy

Adriana Girardi

Brazil

Fernando Giuffrida

Brazil

Romina di Giuseppe

Germany

Liana Del Gobbo

Canada

Ming C. Gong

USA

Arantxa González

Spain

Daniel Gordin

Finland

Philippe Gosset

France

Atsushi Goto

Japan

David Grieve

UK

Hilary Grocott

Canada

Klaus Groschner

Austria

Samuel Grossman

USA

Jian-Wei Gu

USA

Ming Guan

China

Lena Håglin

Sweden

Ganesh V. Halade

USA

Samar M. Hammad

USA

Seung Hyeok Han

South Korea

Yaling Han

China

Kristian Hansen

Norway

Joshua Hare

USA

Karsten Hartvigsen

USA

Toshio Hayashi

Japan

Guoping He

USA

Anthony M. Heagerty

UK

Joerg Heeren

Germany

Daniel Henrion

France

Marit Herder

Norway

Michel P. Hermans

Belgium

Jenny Hernestal-Boman

Sweden

Franz Hessel

Germany

Michael Hill

USA

Katsuya Hirano

Japan

Tsutomu Hirano

Japan

Iren D. Hjellestad

Norway

Jøran Hjelmesæth

Norway

Edith Hohhauser

Israel

Peter Holmquist

Sweden

Stephen P. Hoole

UK

Mads Hornum

Denmark

Policarp P. Hortolà

Spain

Guangpei Hou

Canada

Shengshou Hu

China

Ke Huang

USA

Po-Hsun Huang

Taiwan

Qiaobing Huang

China

Joseph Hung

Austria

Joseph A. Hyder

USA

Borja Ibáñez

Spain

Satoshi Ikeda

Japan

Uichi Ikeda

Japan

Fotios Iliadis

Greece

Toshio Imanishi

Japan

Toru Inami

Japan

Toshihiko Inukai

Japan

San-e Ishikawa

Japan

Shizukiyo Ishikawa

Japan

Nobukazu Ishizaka

Japan

Nobuhiko Ishizuka

Japan

Bruce Ito

USA

Masahiro Itoh

Japan

Luigi Iuliano

Italy

Stephane Jaisson

France

Thomas Jax

Germany

Johan Jendle

Sweden

Myung Ho Jeong

South Korea

Gyorgy Jermendy

Hungary

Weiping Jia

China

Ishwarlal Jialal

USA

Man Hong Jim

Hong Kong

Manuel F. Jiménez-Navarro

Spain

Yan Jinchuan

China

Xia Jing

USA

Odd E. Johansen

Norway

Corinne Jolivalt

USA

Michi Jougasaki

Japan

In Hyun Jung

South Korea

Markus Juonala

Finland

Susanne Kaser

Austria

Naoto N. Katakami

Japan

Akihiko Kato

Japan

Ryuichi Kawamoto

Japan

Mykola Khalangot

Ukraine

Zia Khan

Canada

Francis Kim

USA

Kyoung-Kon Kim

South Korea

Allen B. King

USA

Victoria L. King

USA

Ken Kishida

Japan

Toshihiro Kita

Japan

Antti Kiviniemi

Finland

Colette M. Knight

USA

Daiki Kobayashi

Japan

Katsuhiko Kohara

Japan

Yoshihiro Kokubo

Japan

Jan Komorowski

Poland

Ping Kong

USA

Weimin Kong

USA

Hirokazu Konishi

Japan

Hyun Kook

South Korea

Sietse J. Koopmans

Netherlands

Juraj Koska

USA

Efstathios Kouloridis

Greece

Hanna Kozlowska

Poland

G. V. V. Krishnaveni

India

Anna Kucharska-Newton

USA

Thomas Kunt

Abu Dhabi

Willem van der Laarse

Netherlands

Tahsini Lachin

Iran

Ana Marice Ladeia

Brazil

Solène Le Douairon Lahaye

France

Aipinh Lai

China

Gijs Landman

Netherlands

Chim Lang

UK

Cia-Hin Lau

Singapore

Harold Lazar

USA

Byung-Wan Lee

South Korea

Duk-Hee Lee

South Korea

I-Te Lee

Taiwan

Michael Lehrke

Germany

Markku Lehto

Finland

Andreas Leiherer

Austria

Catherine Lemarie

Canada

Anna Leonardini

Italy

Jonathan Lessick

Israel

Dong Li

USA

Ji Li

USA

Lei Li

USA

Ling Hai Li

China

Qin Li

China

Aris Liakos

Greece

Qiangrong Liang

USA

Bicheng Liu

China

Naifeng Liu

China

Yu Liu

USA

Steven G. Lloyd

USA

Gary Lopaschuk

Canada

Tse-Min Lu

Taiwan

Stephan Lüders

Germany

Joost J. F. P. Luiken

Netherlands

Ida G. Lunde

Norway

Changsheng Ma

China

Xinliang Ma

USA

Yongjie Ma

USA

Rogelio A. Machado

Argentina

Kengo Maeda

Japan

Norikazu Maeda

Japan

Carmen Maldonado-Bernal

Mexico

Safarina S. Malik

Indonesia

Robert Mallet

USA

Licínio Manco

Portugal

Nicolas Mansencal

France

Weil Mao

China

M. Loredana Marcovecchio

Italy

Raffaele Marfella

Italy

Franca Marino

Italy

Rosalind A. Marita

India

Fernanda Marques

Portugal

Seth S. Martin

USA

Carlos Martínez-Salgado

Spain

Luis Masana

Spain

Filipa Mascarenhas-Melo

Portugal

Paulo C. de Freitas Mathias

Brazil

Takayuki Matsumoto

Japan

Bunzo Matsuura

Japan

Anna Vittoria Mattioli

Italy

Paramahamsa Maturu

USA

Michal Mazurek

Poland

Diego Mazzotti

Brazil

Kate McCloskey

Australia

Ross McGeoch

UK

Philip McTernan

UK

Antonie van den Meiracker

Netherlands

Linda Melbin

Sweden

Andreas Melidonis

Greece

Luigi Meneghini

USA

Lampros Michalis

Greece

Takayuki Miki

Japan

Paras Mishra

USA

Tomoya Mita

Japan

Tetsuji Miura

Japan

Masaaki Miyata

Japan

Ryo Miyazaki

Japan

Toru Miyoshi

Japan

Noriyoshi Mizuno

Japan

Ji-Oh Mok

South Korea

Anna V. Möllsten

Sweden

Louis H. Monnier

France

Katsuhito Mori

Japan

Aya Morimoto

Japan

Tatsuya Morimoto

Japan

José M. Mostaza

Spain

Evangeline Motley

USA

Michael Motro

Israel

Mohammed M. Moursi

USA

Andrew J. Murray

UK

Lokhandwala F. Mustafa

USA

Ryoji Nagai

Japan

Naoki Nakagawa

Japan

Kimihiko Nakatani

Japan

Manouchehr Nakhjavani

Iran

Dragana Nikolic

Italy

Göran Nilsson

Sweden

Yanxia Ning

China

Hiroshi Nishi

Japan

Koichi Node

Japan

Jerzy-Roch Nofer

Germany

Gavin Norton

South Africa

Mads Nybo

Denmark

Philippe Obert

France

Eiji Oda

Japan

Michal Odermarsky

Sweden

Anne P. Ofstad

Norway

Hiroshi Ogawa

Japan

Koji Ohashi

Japan

Shinichi Oikawa

Japan

Takashi Ok

Japan

Yosuke Okada

Japan

Yoshihisa Okamoto

Japan

Katashi Okoshi

Brazil

Sukma Oktavianthi

Indonesia

Bjorn Olde

Sweden

Antonio Ribeiro de Oliveira Jr.

Brazil

Altan Onat

Turkey

Michael O'Rourke

Australia

Eduardo Ortega

Spain

Emilio Ortega

Spain

Francisco Ortega

Spain

Carolina Ortega-Azorín

Spain

Anthony O'Sullivan

Australia

Tsuguhito Ota

Japan

Matilde Otero-Losada

Argentina

Maciej Owecki

Poland

Barry Palmer

New Zealand

Bradley Palmer

USA

Carlo Palombo

Italy

Sandeep V. Pandit

USA

Juan A. Paniagua

Spain

Giuseppe Papa

Italy

Athanasia Papazafiropoulou

Greece

Klaus G. Parhofer

Germany

Hyeong-Kyu Park

South Korea

Jeong Hyun Park

South Korea

James Pearson

USA

Alberto Penas-Steinhardt

Argentina

Wenhui Peng

China

Frederik Persson

Denmark

Hitesh Peshavariya

Australia

Ligia Petrica

Romania

John Petrie

UK

Marija Pfeifer

Slovenia

Antonello Pileggi

USA

Amos Pines

Israel

Frank Pistrosch

Germany

David Polidori

USA

Pavel Poredos

Slovenia

Nick Pullen

UK

Jiancheng Qi

Australia

Yong Fen Qi

China

Waqas Qureshi

USA

Eric Raddatz.

Switzerland

Tamas Radovits

Germany

Sergio Raposeiras-Roubín

Spain

Bjorn Rathsman

Sweden

Susana Ravassa

Spain

Itamar Raz

Israel

Alejandro Rebolledo

Argentina

Zeljko Reiner

Croatia

Flavio Reis

Portugal

S. Reitsma

Netherlands

Marie-Ange Renault

France

Markus Resch

Germany

Ravi Retnakaran

Canada

Sirimon Reutrakul

Thailand

Eun-Jung Rhee

South Korea

Cristobal Richart

Spain

Walter Riesen

Switzerland

Esther van 't Riet

Netherlands

Dena Rifkin

USA

Arne Ring

Germany

Arne Ring

UK

Manfredi Rizzo

Italy

Denise Roche

UK

Bruno Rodrigues

Brazil

Oliva Mejía Rodríguez

Mexico

Greet Roef

Belgium

Camila M. Rosa

Brazil

Paolo Rossetti

Italy

Ina-Maria Rückert

Germany

Lars Ryden

Sweden

Charumathi Sabanayagam

Singapore

Bernard Saiag

France

Yasushi Saito

Japan

Masaya Sakamoto

Japan

Federico Salamone

Italy

Carlos A. Aguilar Salinas

Mexico

Sanjay Samy

USA

José L. Sánchez-Quesada

Spain

Alice Santos-Silva

Portugal

Marta Sarkozy

Hungary

Masataka Sata

Japan

Les Satin

USA

Tatsuya Sato

Japan

Jo Satoh

Japan

Judy Savige

Australia

Takahiro Sawada

Japan

Peter P. Sayeski

USA

Ludger Scheja

Germany

Karin Schenck-Gustafsson

Sweden

Peter Schmid

Germany

Roland Schmieder

Germany

Arthur Scholte

Netherlands

Alexandra Scholze

Denmark

Vera Schrauwen-Hinderling

Netherlands

Aletta Schutte

South Africa

Rudolph Schutte

South Africa

David Sedmera

Czech Republic

Elio di Segni

Israel

Ingebjørg Seljeflot

Norway

Anne-Marie L. Seymour

UK

Marina Shargorodsky

Israel

Neeru Sharma

USA

Alexander Sheehy

USA

Joseph Shemesh

Israel

Shigeki Shibahara

Japan

Michio Shimabukuro

Japan

Hiroaki Shimokawa

Japan

Joon-Han Shin

South Korea

Kou-Gi Shyu

Taiwan

Alfonso Siani

Italy

Rosa Sicari

Italy

Niina S. Siitonen

Finland

Edson da Silva

Brazil

Marcia Ferreira da Silva

Brazil

Amit S. Singh

India

Satyesh Sinha

USA

Sandhya Sitasawad

India

Ake Sjoholm

Sweden

Elsayed Z. Soliman

USA

David P. Sonne

Denmark

James R. Sowers

USA

Janet Sparks

USA

Slavica Spasic

Serbia

Alessandro Squizzato

Italy

Bart Staels

France

Eberhard Standl

Germany

Doris Stöckl

Germany

Mark W. Stolar

USA

Huabo Su

USA

Savitha Subramanian

USA

Deepa Sumukadas

UK

Ki Chul Sung

South Korea

Velipekka Suominen

Finland

Kurt Svardsudd

Sweden

Abd Tahrani

UK

Yasuo Takahashi

Japan

Noriaki Takama

Japan

Kouichi Tamura

Japan

Mengxiong Tang

China

Edite Teixeira-Lemos

Portugal

Costas C. Tellis

Greece

Tamio Teramoto

Japan

Chris Terpening

USA

Frank Thies

UK

Eduardo Tibirica

Brazil

Sara Tomei

Qatar

Marek Treiman

Denmark

Hung-Fat Tse

Hong Kong

Panayoula Tsiotra

Greece

Kunihiro Tsuchida

Japan

Aage Tverdal

Norway

John Ussher

Canada

Gian Ussia

Italy

Tone G. Valderhaug

Norway

Tony Velkov

USA

Teresa Villarreal

Mexico

Mohan Viswanathan

India

Massimo Volpe

Italy

Jun Wada

Japan

Robert Wagner

Germany

Werner Waldhausl

Austria

Daowen Wang

China

Guoping Wang

China

Jiguang Wang

China

Zhen Wang

USA

Sasiwarang Wannamethee

UK

Hirotaka Watada

Japan

Kenchi Watanabe

Japan

Karol Watson

USA

Eugene Weinberg

USA

Charles Weissman

Israel

Jan Westerink

Netherlands

Nicolas Wiernsperger

France

Anna Witasp

Sweden

Paulus Wohlfart

Germany

Johann Wojta

Austria

G. William Wong

USA

Jie Wu

USA

Yen-Wen Wu

Taiwan

Yong Xia

USA

Zhengyuan Xia

Hong Kong

Liang Xiao

China

Aimin Xu

Hong Kong

Sam Xu

UK

Xiulong Xu

USA

Daisuke Yabe

Japan

Masanori Yamamoto

Japan

Minako Yamaoka-Tojo

Japan

Hideyuki Yamawaki

Japan

Peixin Yang

USA

Yoshinari Yasuda

Japan

Jianping Ye

USA

Shui Q. Ye

USA

Yicong Ye

China

Bu B. Yeap

Australia

Joseph Yeboah

USA

Derek Yellon

UK

Kai Hang Yiu

Hong Kong

Koutaro Yokote

Japan

Shinji Yokoyama

Japan

Byung-Woo Yoon

South Korea

Demin Yu

China

Katherine Yutzey

USA

Fernanda S. Zamo

Brazil

Hany A. Zayed

UK

Christine Zelenak

Canada

Ting Zhao

USA

Zhi-Qing Zhao

USA

Lemin Zheng

China

Lovro Ziberna

Italy

Andreas Zirlik

Germany

Tali Zitman-Gal

Israel

Theodoros Zografos

Greece

Andrea Zsombok

USA

Coert C. J. Zuurbier

Netherlands

